# Supplementary material for: Condensation of preformed charge density waves in kagome metals
Source: Nat Commun. 2023 Nov 11;14:7309. doi: 10.1038/s41467-023-43170-w (PMC10640577; doi:10.1038/s41467-023-43170-w)
Supplement: Supplementary file 1 — Supplementary Information [file 41467_2023_43170_MOESM1_ESM.pdf]

Supplementary Information: Condensation of preformed charge density waves in kagome metals

Changwon Park and Young-Woo Son
Korea Institute for Advanced Study, Seoul 02455, Korea

1. LATTICE PARAMETERS AND CDW FORMATION ENERGIES

In Table I, we provide detailed first-principles calculation results for structural parameters of various charge density wave (CDW) states as well as their energetics.

2. ENERGETICS OF 4-STATES STACKING

We consider the following 4-states Potts model on the layers of triangular lattice,

$$\mathcal{H} = \sum_{\langle i,j\rangle,\alpha} J_{\parallel}\delta(s_{i,\alpha}, s_{j,\alpha}) + \sum_{i,\alpha} J_{\perp n}\delta(s_{i,\alpha}, s_{i,\alpha+n}), \quad (1)$$

where $s_{i,\alpha}$ is four-states spin variable at i-th site of α-th layer, while $J_{\parallel} < 0$ and $J_{\perp n}$ are the nearest-neighbor in-plane interaction and out-of-plane n-th nearest-neighbor interactions, respectively. We assume $|J_{\parallel}| \gg |J_{\perp n}|$, and $|J_{\perp n}|$ becomes smaller as n becomes larger. For the simplified analysis, we further assume $|J_{\perp 1}|$ is larger than $|J_{\perp 2}| + |J_{\perp 3}|$.

When $J_{\perp 1} > 0$, the ground state becomes trivially ferromagnetic and the stacking order can be represented as (A)(A)(A)$\cdots$. If $J_{\perp 1} < 0$, to uniquely determine the ground states, a finite $J_{\perp 2}$ should be introduced. When $J_{\perp 2} < 0$, second-neighboring layers favors to have the same phase with each other, and the ground state stacking order becomes (AB)(AB)(AB)$\cdots$ ($2\times2\times2$ stacking).

Supplementary Table I. *Ab initio* calculation results for lattice parameters (in Angstrom) of a and c along in-plane and out-of-plane direction, respectively, and CDW formation energies of E_{CDW} (in meV/formula unit) for various CDW phases shown in Supplementary Fig. 1**d**. Numbers in parentheses are from experiments. In the last row, E_{CDW}'s using our interatomic potentials method are shown.

	lattice parameter		E_{CDW}		
	a	c	$2\times2\times1$	$2\times2\times2$	$2\times2\times4$
KV_3Sb_5	5.412 (5.482)[a]	8.886 (8.958)[a]	−4.2	−7.6	−7.6
RbV_3Sb_5	5.414 (5.472)[b]	9.086 (9.073)[b]	−5.5	−8.8	−8.9
CsV_3Sb_5	5.437 (5.480)[c]	9.334 (9.320)[c]	−12.1	−14.4	−14.7
CsV_3Sb_5[d]			−11.8	−15.9	−15.7

[a] Ref. [1] [b] Ref. [2] [c] Ref. [3] [d] Our interatomic potential

For $J_{\perp 2} > 0$, we again need $J_{\perp 3}$ to uniquely determine the ground states. The $J_{\perp n}$-dependence of ground states are summarized in Supplementary Fig. 1(a). Especially for systems with $J_{\perp 1} > 0$ and $J_{\perp 2} < 0$, if the temperature (T) becomes comparable with $J_{\perp 2}$, other low-energy stacking structures such as (ABC)(ABC)$\cdots$ and (ABCD)(ABCD)$\cdots$ shown in Supplementary Fig. 1(b) can be locally stabilized thanks to the entropic effect.

3. FINITE-TEMPERATURE PHONON SPECTRUM OF CsV_3Sb_5

In Supplementary Fig. 2, we display our simulation results for phonon dispersions with various temperature along different symmetric paths in Brillouin zone (BZ) of the pristine unitcell.

In Supplementary Fig. 3, we also compare temperature-dependent phonon spectral amplitudes from $S_{\rho\rho}(\mathbf{k},\omega;T)$ at different momentum as well as with different simulation time scales. We note that, as shown in Supplementary Figs. 3 (b) and (d) for $12\times12\times12$ MD simulation at $T = 140$ K, soft phonon mode at L point still remains, confirming the presence (absence) of a soft mode at $L(\Gamma)$ is not affected by the domain fluctuations. In Supplementary Figs. 3 (b) and (d), slow dynamics of CDW phase fluctuations are included in MD trajectories and they are manifested as peaks near zero frequency at $T_{\mathrm{CDW}} < T < T^{*}$.

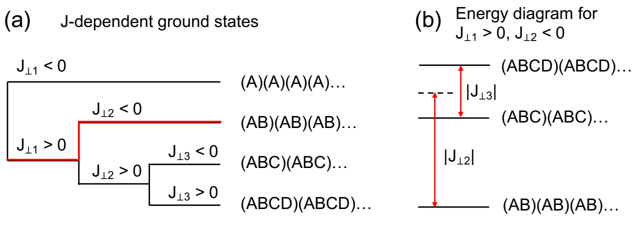

Supplementary Fig. 1. Ground stacking configuration for various out-of-plane interactions $J_{\perp n}$ (nth neighboring layers). $|J_{\perp 1}| > |J_{\perp 2}| + |J_{\perp 3}|$ and $|J_{\perp 2}| > |J_{\perp 3}|$ is assumed. (b) Energy diagram of low energy stacking orders for $J_{\perp 1} > 0$ and $J_{\perp 2} < 0$ (red line in (a)).

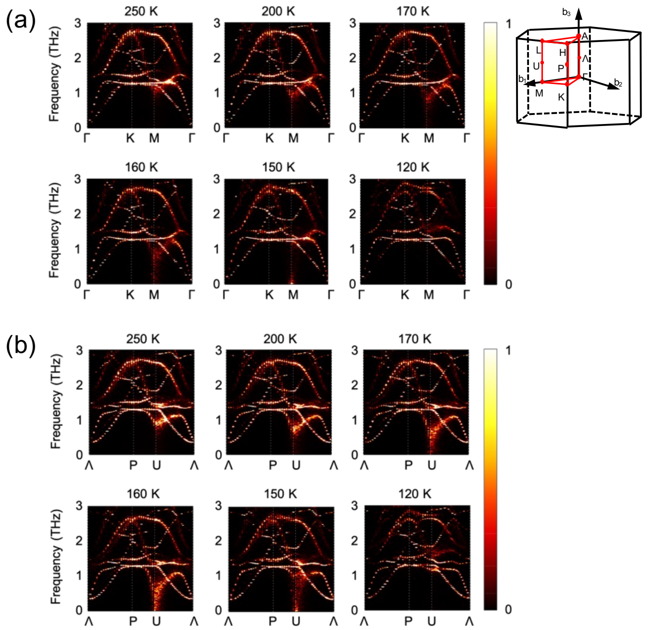

Supplementary Fig. 2. Finite temperature phonon spectrum from $S_{\rho\rho}(\mathbf{k}, \omega; T)$ based on MD simulations of $60 \times 60 \times 12$ supercell for 0.4 nanoseconds. The color codes of the intensities are the same as Fig. 2c. The phonon spectra along symmetric lines on (k_x, k_y) plane with (a) $k_z = 0$ and (b) $k_z = \frac{1}{4} b_z$, respectively. b_z is the out-of-plane reciprocal lattice constant. BZ and high-symmetric points are shown on the right panel.

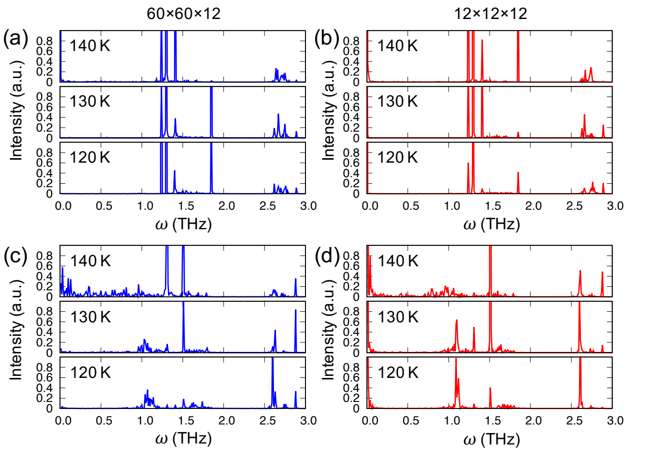

Supplementary Fig. 3. $S_{\rho\rho}(\mathbf{k}, \omega; T)$ when $\mathbf{k}$ is at (a)-(b) Γ- and (c)-(d) L-point, respectively. (a) and (c) is obtained from MD simulation of $60 \times 60 \times 12$ supercell for 0.4 nanoseconds, and (b) and (d) from $12 \times 12 \times 12$ supercell for 12 nanoseconds.

4. SIZE EFFECT

To confirm that the sign changing events in Fig. 3**a** of the main manuscript are not just a local fluctuation but a phase flip of whole layer, the events between 7 and 8 nanosecond are presented in Supplementary Fig. 4. Each panel is obtained from the snapshot of molecular dynamics (MD) simulations taken every 50 femtoseconds. Here, 2×2 unit cells and the amplitudes m_1 for the cells in the panel are presented with the circles and their graded colors, respectively. The phase flip in a layer follows a typical nucleation and growth mechanism; phase flips at 2×2 cells happen to coalesce to form a domain (black circle) and the growth/merge (ellipse) of the domains results in the phase flip of the whole layer. This implies that the finite size effect makes the rate of phase flips to be somewhat overestimated because domains can have more chances of merging while its melting probability is ignored when the size is larger than the simulation cell. Nevertheless, the thermodynamic stability of domain formation is still valid because the initial nucleation process is insensitive to the boundary condition.

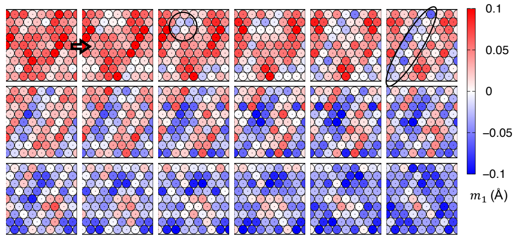

Supplementary Fig. 4. Typical nucleation and growth mechanism leading to CDW phase flips. The snapshots of 12×12 supercells are taken every 50 femtoseconds and presented from the left top to the right bottom as increasing the time step. The circles and their colors represent 2×2 supercells and the amplitudes m_1, respectively. The domain starts from cluster (large circle) and grows/merges into one-dimensional strip (ellipse) and finally, the whole layer flips its phase.

5. EIGENMODE DECOMPOSITION AND CORRELATION ANALYSIS OF CDW

To investigate the fluctuation dynamics of preformed CDW, the instantaneous local CDW order should be distinguished from trivial atomic vibrations. We found the $2 \times 2 \times 2$ CDW in CsV_3Sb_5 can be accurately described with a few low-energy eigenmodes and 94% of its atomic displacement are projected to three symmetrically equivalent lowest eigenmodes at M points (q_M^1, q_M^2, q_M^3 in Supplementary Fig. 5). For CsV_3Sb_5, all q_M^i ($i = 1, 2, 3$) are real such that their phases can be absorbed to amplitude m_i ($i = 1, 2, 3$) as a sign, and we will use three signed amplitudes of m_i as a descriptor for a local CDW order in $2 \times 2 \times 1$ supercell as shown in Fig. 1**c** of the main manuscript. Note we choose only one of the time-reversal pair of eigenmodes because their phases are complex con-

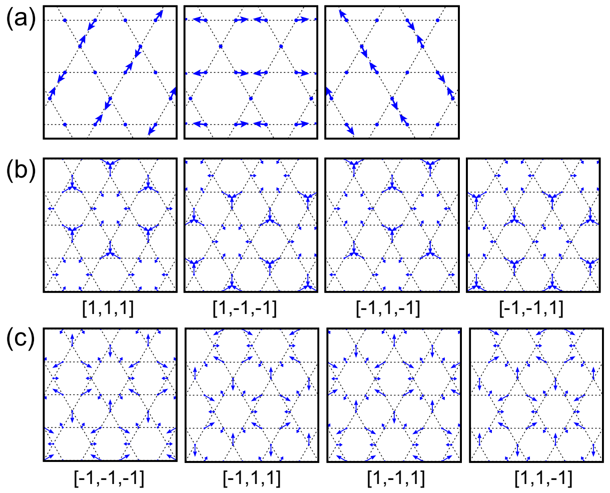

Supplementary Fig. 5. (a) From left to right panels, eigenvectors of three eigenmodes of $q_M^i (i = 1, 2, 3)$ at M points. Blues dots are kagome lattice points and arrows denotes atomic displacements. Linear combinations of three q_M^i or $m_1 q_M^1 + m_2 q_M^2 + m_3 q_M^3$ denoted as $[m_1, m_2, m_3]$ form (b) inverse star of David (iSOD) pattern or (c) star of David (SOD) pattern. We note that when a product of three coefficients, $m_1 m_2 m_3$, is positive (negative), the resulting configuration becomes iSOD (SOD).

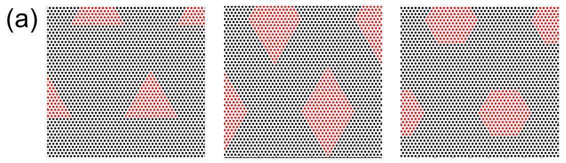

(b)

shape	$A(n)$	$L(n)$	$E(n) = A(n)J_\perp + L(n)J_\parallel$
triangle	$\frac{(n+1)(n+2)}{2}$	$3n + 6$	$\frac{1}{2}J_\perp n^2 + \left(\frac{3}{2}J_\perp + 3J_\parallel\right)n + J_\perp + J_\parallel$
rhombus	n^2	$4n + 2$	$J_\perp n^2 + 4J_\parallel n + 2J_\parallel$
hexagon	$3n^2 + 3n + 1$	$6n + 6$	$3J_\perp n^2 + (3J_\perp + 6J_\parallel)n + J_\perp + 6J_\parallel$

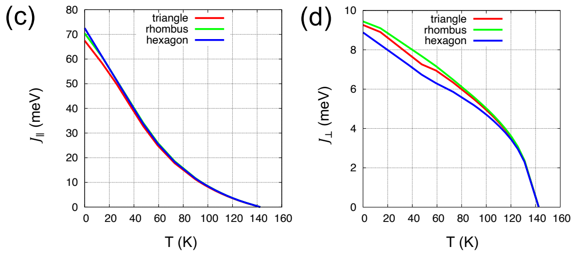

Supplementary Fig. 7. From the left, triangular, rhombic and hexagonal CDW domain on triangular lattices. Each lattice point corresponds 2×2 supercell of $A\mathrm{V_3Sb_5}$ and CDW phases are distinguished by red and black colors. (b) Areas $A(n)$, number of neighboring cells $L(n)$ and energy $E(n)$ of the three domains. n denotes the length of side. (c) intralayer and (d) interlayer interaction parameters calculated from the least square fitting of domain energies.

jugate of each other from the constraint that atomic displacements are real. Three q_M^i $(i = 1, 2, 3)$ are shown in Supplementary Fig. 5(a) and 2×2 CDW displacement patterns composed of the linear combinations of them are in Supplementary Fig. 5(b) and (c). When the three amplitudes are the same in the magnitudes, depending on the sign of $m_1 m_2 m_3$, they have either star-of-David or inverse star-of-David pattern.

Typical time evolutions of m_1 are demonstrated in Supplementary Fig. 6(a) for systems with preformed CDW at 120 K (blue), without CDW at 250 K(red) and with both preformed CDW and slow domain fluctuations at 140 K (green), where the preformation of CDWs can be identified as nonzero average value of m_1.

The correlation and dynamics of local 2×2 CDW are quantified using Pearson correlation coefficients (PCC) r_{xy} defines as

$$r_{xy} = \frac{\sum_{i=1}^{n}(x_i - \bar{x})(y_i - \bar{y})}{\sqrt{\sum_{i=1}^{n}(x_i - \bar{x})^2}\sqrt{\sum_{i=1}^{n}(y_i - \bar{y})^2}} \quad (2)$$

where $\bar{x}$ denotes the average of x and $x = m_i(\mathbf{R}, t)$, $y = m_j(\mathbf{R} + \Delta\mathbf{R}, t + \Delta t)$. By sufficiently sampling $\mathbf{R}$ and t, the correlation function $r_{ij}(\Delta\mathbf{R}, \Delta t)$ can be defined as PCC of x and y. Supplementary Figures 6(b) and (c) shows the schematic scatter plots of $m_1(\mathbf{R}, t)$ and $m_1(\mathbf{R}, t + \Delta t)$ and corresponding temporal correlation function $r_{11}(\Delta\mathbf{R} = 0, \Delta t) \equiv r_{11}(\Delta t)$, respectively. In common, $r_{11}(\Delta t)$ has oscillations with typical phonon frequency. For a system with CDW (blue), the asymptotic value of $r_{11}(\Delta t)$ remains finite while it decays fast to zero (red) if no static CDW exists. If there is slower dynamics such as sign flips of m_i (green), it is manifested

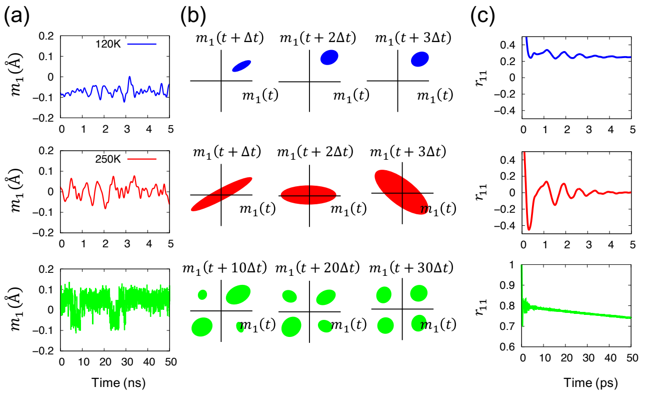

Supplementary Fig. 6. (a) Time evolutions of the amplitudes m_1 of eigenmode q_M at T = 120 K (blue), 250 K (red) and 140 K with domain flipping dynamics (green). For former two cases, domain flip dynamics cannot be captured due to short simulation time. (b) schematic scatter plots between $m_1(\mathbf{R}, t)$ and $m_1(\mathbf{R}, t + \Delta t)$. Large sampling density regions are colored. (c) Pearson correlation coefficients $r_{11}(\Delta\mathbf{R} = 0, \Delta t)$.

as the slow decay of $r_{11}(\Delta t)$, which is a good measure of phase flip rate.

6. FORMATION ENERGY OF CDW DOMAINS

We found that the considered CDW domain here is a local minimum in neither our interatomic potential nor thermally averaged potential. So, there seems to be no energy barrier to stabilize the finite-sized CDW domains. Therefore, during the optimization step, we fixed vanadium atoms in a CDW domain and were able to stabilize various shapes of CDW domains. We also have checked that fixing Sb atoms gives the similar domain energy within 5%.

From the formation energy of CDW domain, the intra- and inter-layer interaction parameters $J_{\|}$ and $J_{\perp}$ are calculated as follows. We assume the formation energy E can be accurately approximated as $E = AJ_{\perp} + LJ_{\|}$ where A and L are the area and circumference (more accurately, the number of neighboring 2×2 cells) of the domain. Supplementary Figure 7(a) is the three types of CDW domains we have considered and Supplementary Fig. 7(b) summarizes the geometry and the energy of the domains. For each shape of domain, we varied the side length n from 5 to 12 and the calculated domain energies are least-square-fitted with $E(n)$. The fitted $J_{\|}$ and $J_{\perp}$ are plotted in Supplementary Fig. 7(c) and (d), respectively, where the shape dependences are negligible.

As T increases, the ratio between inter- and intralayer interaction parameter $J_{\perp}/J_{\|}$ is also sharply increases as shown in Supplementary Fig. 8(a). One of the reason for the different temperature dependence of $J_{\|}$ and $J_{\perp}$ is the different behaviors of domain wall. Supplementary Figure 8(b) shows a rhombic CDW domain of CsV_3Sb_5 at 0 K drawn with the same scheme as Supplementary Fig. 4. The line profiles along the arrow are compared for the case of $T = 0$ K and $T = 120$ K in Supplementary Fig. 8(c) and (d), respectively, and we can see the width of domain wall is extended to reduce the formation energy when the temperature is high (120 K). On the contrary, the width of domain wall along the out-of-plane direction cannot exceed one unitcell so that no degree of freedom is available to reduce the formation energy for $J_{\perp}$.

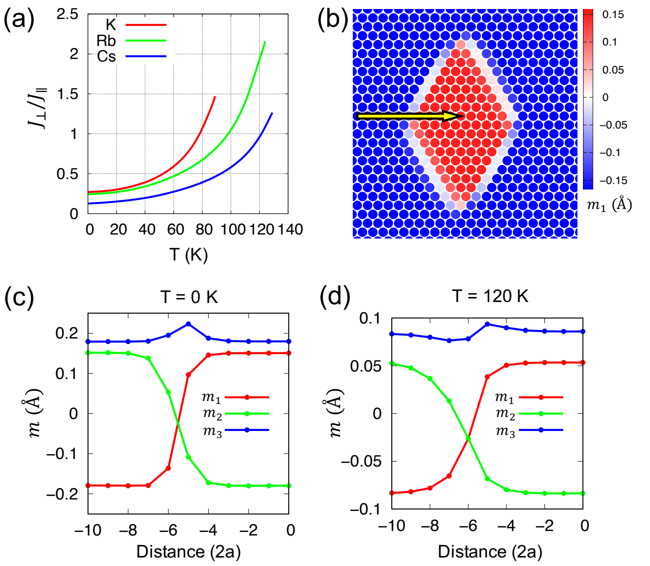

Supplementary Fig. 8. (a) Temperature-dependent ratio between inter- and intralayer interaction parameters of KV_3Sb_5 (red), RbV_3Sb_5 (green) and CsV_3Sb_5 (blue). (b) Rhombic CDW domain of CsV_3Sb_5 at 0 K. Each circle is a 2×2 supercell and m_1 of the cell is represented with graded colors as shown in the color bar. (c) Line profile indicated by the arrow in (b). Gradual CDW phase changes at the domain wall are reflected in the sign changes of m_1 and m_2. The unit of horizontal axis is $2a$ (a is in-plane lattice constant of CsV_3Sb_5). (d) The same line profile at $T = 120$ K. The width of domain wall corresponding the slope of m_1 (or m_2) becomes increased.

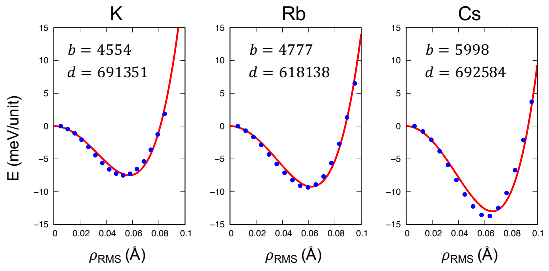

Supplementary Fig. 9. Total energy changes of CDWs in AV_3Sb_5. From left to right panels, A = K, Rb and Cs, respectivley. Horizontal axes of ρ_{RMS} are root mean squares of atomic displacements scaled by maximum values. Blue dots are from DFT and red lines are least square fits on $E = -b\rho^2 + c\rho^3 + d\rho^4$.

7. SCALED INTERATOMIC POTENTIALS

In Supplementary Fig. 9, we display total energy changes of $2 \times 2 \times 2$ CDWs of AV_3Sb_5 when ground state CDW distortions are linearly scaled. The computed DFT total energies are fit with $E = -b\rho^2 + c\rho^3 + d\rho^4$. Fitted vales of c are lesser than 0.003 for all cases and are not shown here. As shown in Supplementary Fig. 9, the interatomic potential of CsV_3Sb_5 well reproduces the profile so that we constructed the interatomic potential of KV_3Sb_5 and RbV_3Sb_5 by scaling the force constants of CsV_3Sb_5.

[1] B. R. Ortiz, L. C. Gomes, J. R. Morey, M. Winiarski, M. Bordelon, J. S. Mangum, I. W. H. Oswald, J. A. Rodriguez-Rivera, J. R. Neilson, S. D. Wilson, E. Ertekin,

T. M. McQueen, and E. S. Toberer, New kagome prototype materials: discovery of KV_3Sb_5, RbV_3Sb_5, and CsV_3Sb_5, Phys. Rev. Mater. **3**, 094407 (2019).

[2] S. Cho, H. Ma, W. Xia, Y. Yang, Z. Liu, Z. Huang, Z. Jiang, X. Lu, J. Liu, Z. Liu, J. Li, J. Wang, Y. Liu, J. Jia, Y. Guo, J. Liu, and D. Shen, Emergence of new van hove singularities in the charge density wave state of a topological kagome metal RbV_3Sb_5, Phys. Rev. Lett. **127**, 236401 (2021).

[3] A. A. Tsirlin, P. Fertey, B. R. Ortiz, B. Klis, V. Merkl, M. Dressel, S. D. Wilson, and E. Uykur, Role of Sb in the superconducting kagome metal CsV_3Sb_5 revealed by its anisotropic compression, SciPost Phys. **12**, 049 (2022).
